# A Novel Functional Domain of Tab2 Involved in the Interaction with Estrogen Receptor Alpha in Breast Cancer Cells

**DOI:** 10.1371/journal.pone.0168639

**Published:** 2016-12-19

**Authors:** Stefania Reineri, Silvia Agati, Valentina Miano, Monica Sani, Paola Berchialla, Laura Ricci, Andrea Iannello, Lucia Coscujuela Tarrero, Santina Cutrupi, Michele De Bortoli

**Affiliations:** 1 Department of Clinical and Biological Sciences, University of Turin, Turin, Italy; 2 Bioindustry Park Silvano Fumero, Colleretto Giacosa, Turin, Italy; 3 Center for Molecular Systems Biology, University of Turin, Turin, Italy; 4 CNR, i.c.r.m. Institute of Chemistry of Molecular Recognition, Milan, Italy; Seconda Universita degli Studi di Napoli, ITALY

## Abstract

Tab2, originally described as a component of the inflammatory pathway, has been implicated in phenomena of gene de-repression in several contexts, due to its ability to interact with the NCoR corepressor. Tab2 interacts also with steroid receptors and dismisses NCoR from antagonist-bound Estrogen and Androgen Receptors on gene regulatory regions, thus modifying their transcriptional activity and leading to pharmacological resistance in breast and prostate cancer cells. We demonstrated previously that either Tab2 knock-down, or a peptide mimicking the Estrogen Receptor alpha domain interacting with Tab2, restore the antiproliferative response to Tamoxifen in Tamoxifen-resistant breast cancer cells. In this work, we map the domain of Tab2 responsible of Estrogen Receptor alpha interaction. First, using both co-immunoprecipitation and pull-down with recombinant proteins, we found that the central part of Tab2 is primarily responsible for this interaction, and that this region also interacts with Androgen Receptor. Then, we narrowed down the essential interaction region by means of competition assays using recombinant protein pull-down. The interaction motif was finally identified as a small region adjacent to, but not overlapping, the Tab2 MEKK1 phosphorylation sites. A synthetic peptide mimicking this motif efficiently displaced Tab2 from interacting with recombinant Estrogen Receptor alpha in vitro, prompting us to test its efficacy using derivatives of the MCF7 breast carcinoma cell lines that are spontaneously resistant to Tamoxifen. Indeed, we observed that this mimic peptide, made cell-permeable by addition of the TAT minimal carrier domain, reduced the growth of Tamoxifen-resistant MCF7 cells in the presence of Tamoxifen. These data indicate a novel functional domain of the Tab2 protein with potential application in drug design.

## Introduction

The Tab2 protein (also known as TGF-β activated kinase 1/MAP3K7 binding protein 2) has been implicated in the mechanisms of resistance to steroid antagonists in breast and prostate cancer, contributing an additional piece of the jigsaw to the long-standing concept of cross-talk between inflammation and hormonal response, as well as between inflammation and cancer. Indeed, the Tab2 protein was originally described as a component of the TNFα/TGF-β and inflammatory cytokines transduction pathways [1,2]. Tab2 was firstly identified as an adaptor protein in the cytoplasm linking TAK1 and TRAF6 in the interleukin-1β (IL-1β) signaling pathway [1], suggesting that activation of the NF-kB and MAPK cascades by IL-1β involves the formation of a TRAF6-Tab2-TAK1 complex. In this complex, TRAF6 is autopolyubiquitinated with Lys-63-linked ubiquitin chains, through which it interacts with Tab2, leading to the binding and activation of TAK1 and subsequently the activation of IKK and NF-kB [3]. Thus, Tab2 ubiquitination mediated by TRAF6 appears to play an important role in TAK1 activation in IL-1β signal transduction [4].

In addition to its role as adaptor in the cytokine signaling pathway, Tab2 displays regulatory roles in transcriptional repression, in conjunction with the NCoR corepressor complex, in different contexts such as NF-kB regulated genes [5], astrocyte-specific genes in neuronal precursors [6] and prostate and breast cancer cells, where Tab2 mediates reversion of steroid receptor antagonists effects in response to inflammation [7,8].

First, in neurodegeneration a model has been proposed, in which Tab2 is recruited to NF-kB regulated genes by an interaction with the Bcl3 protein, acting as a bridge linking NF-kB to nuclear coregulators [5]. In response to IL-1β signaling, the stable Tab2/HDAC3/NCoR corepressor complex, bound to p50 target genes, undergoes translocation to the cytoplasm, resulting in the recruitment of Tip60-containing coactivator complex on NF-kB target genes. In this model, Tab2 has a dual role in response to IL-1β: it acts both to contribute to de-repression of p50-dependent transcription unit (its nuclear action) and to activate the NF-kB pathways (its cytoplasmic function), as previously demonstrated by Takaesu et al. [1].

The second context, proposed by Sardi and colleagues, involved the regulation of the timing of astrogenesis in the developing brain [6]. The E4ICD cytoplasmic domain of ErbB4, released by presenilin after activation by the ligand neuregulin-1, is able to interact with Tab2 in a specific manner and this is dependent on the tyrosine kinase activity of the ErbB4 fragment. Tab2, acting as an adaptor molecule, forms a ternary E4ICD/Tab2/NCoR complex that could be detected in lysates from neuregulin-1-stimulated cells. This complex undergoes translocation to the nucleus, where it targets several glial genes to transcriptional repression, which is required for the differentiation of neuronal precursor cells into astrocytes.

The third model involves the interaction between Tab2 and the Androgen Receptor (AR) or the Estrogen Receptor alpha (ERα) in mediating reversion of steroid receptor antagonists effects in response to inflammation [7,8]. Studies on antiandrogen-resistant prostate cancer cells showed that IL-1β induces phosphorylation of Tab2 engaged in the nucleus with NCoR complexes, allowing Tab2 to translocate to the cytoplasm together with NCoR, thus dismissing repression from androgen responsive genes and functionally converting antiandrogenic compounds to androgenic [7]. In response to IL-1β, the MAPK kinase kinase 1 (MEKK1) phosphorylates Tab2, unmasking its nuclear export signal sequence (NES). Thus, the NCoR/Tab2 complex is dismissed from AR, and coactivators can associate, instead. As a consequence, the antiandrogenic drug Bicalutamide is switched from repressing AR transcriptional activity to stimulating it [7]. Interestingly, suppression of Tab2 resulted in reversal of IL-1β effect, demonstrating that Tab2 is not essential to transcriptional repression by AR. A similar mechanism was observed in MCF7 breast cancer cells in response to Tamoxifen (Tam). Tab2 is recruited to ERα- or AR-responsive genes through interaction with an L/HX7LL conserved motif in the N-terminal domain of sex steroid receptors proteins (AR, ERα, and Progestin receptor (PgR)). A peptide mimicking this region reverts IL-1β induced NCoR dismissal when microinjected in prostate cancer cell nuclei [7].

We demonstrated previously that spontaneously Tam-resistant (TamR) derivatives of the MCF7 breast carcinoma cell line have constitutively phosphorylated Tab2, and knocking-down Tab2 expression by siRNA is sufficient to restore the antiproliferative response to Tam [8], thus implying constitutive activation of Tab2 in endocrine resistance. A region in the central part of Tab2 (CC domain and adjacent regions, see below) contain several Serines and Tyrosines that can be phosphorylated through different pathways, involving MEKK1 and p38 in response to cytokines and EGF [5,9–11]. Moreover, mutations of the MEKK1 gene were also found associated to resistance to aromatase inhibitors [12], involving Tab2 in a more general mechanism of pharmacological resistance. We have also shown that a cell-permeable peptide mimicking aa. 4–17 of hERα [7] abrogates Tab2/ERα interaction *in vitro* and restores Tam response in TamR cells [8].

The interaction between Tab2 and NCoR has been roughly mapped: it involves the repressor domain I of NCoR and the N-terminal region of Tab2 (aa 1–628) [5,6]. Tab2 (and its closely related protein Tab3) contain an N-terminal CUE domain (Cue1-homolog) that includes Phenylalanine and Proline (FP) residues essential for the direct binding of monoubiquitin. In the C-terminal half, other characterized parts include a coiled-coil domain (CC), involved in the interaction with TAK1, and a zinc-finger (NZF) domain also called nuclear protein localization 4 (Npl4) or zf-RanBP domain, involved in polyubiquitin binding [13]. Tab2 and Tab3 bind preferentially to lysine 63-linked polyubiquitin chains through this highly conserved NZF domain, and in fact mutations to NZF abrogate polyubiquitin chain binding together with Tab2/Tab3 ability to activate TAK1 and IKK [14].

Although Tab2 interaction with ERα and AR has been clearly established [7,8], very little is known about the Tab2 domain involved, with the exception of its rough localization to the C-terminal half. More detailed mapping would be an important issue, because it will help shedding light on the exact mechanism of Tab2 recruitment and dynamics within the corepressor complex. Tab2 acts as “molecular beacon” integrating nuclear transcriptional response of different signaling pathways and impinges upon molecular mechanisms that are relevant for clinics and pharmacological intervention, as exemplified by Tam or Bicalutamide or the neurodegenerative context.

In this paper we describe the fine mapping of Tab2 domain involved in ERα interaction and show that a cell-permeable mimic peptide can reverse Tam resistance in MCF7-TamR cells in culture.

## Materials and Methods

### Chemicals and antibodies

4-hydroxytamoxifen (4OHT) was purchased from Sigma-Aldrich. Polyclonal anti-ERα antibody (H-184), anti-MBP (C-18) and anti-NFkB-p65 antibodies were from Santa Cruz Biotechnology. Monoclonal antibody anti-Flag and polyclonal antibody anti-Tab2 (491–505) were from Sigma-Aldrich. Polyclonal anti-epitope T7 antibody was purchased from Abcam. Recombinant human ERα was purchased from Life Technologies. The Tab2 peptides were synthesized by microwave-assisted synthetic protocols and their analysis and purification were carried out by analytical and semi-preparative reversed phase high performance liquid chromatography (RP-HPLC). The TAT-Tab2 peptides were purchased from the Peptide Facility of CRIBI (Biotechnology Center) in Padua, Italy.

### Plasmids, mutants and fragments

The pCMV-T7 containing the full length hTab2 cDNA, the p3XFLAG-CMV containing the hAR cDNA and the pGEX-JDK containing hERα (1–45) were generous gifts from Prof. MG Rosenfeld (UCSD, La Jolla, CA). The hERα expression vector pHEGO and the p3XFLAG-CMV containing the hERα cDNA were generous gifts from Prof. P. Chambon [15] and Prof. A Weisz (University of Naples, Italy), respectively. The cDNA encoding hERα (1–60) was amplified by PCR and cloned into pCMV-p65 vector. The cDNA encoding human Tab2 full-length and all the Tab2 fragments (Δ CUE Tab2, Δ sinoS Tab2, 406-531Tab2, Δ NZF Tab2, Δ NZFCC Tab2, Δ NZFCCSS Tab2, and 1–350 Tab2) were amplified by PCR and cloned into pMALc2 vector. All constructs were verified by automated DNA sequencing. The cDNAs encoding the 6 partially overlapping fragments whose sequence has been designed on the sequence of the Tab2 central fragment 406–531 (Table 1) were amplified by PCR and used to *in vitro* transcribe and translate (TnT) all fragments using the TNT^®^ T7 Quick Coupled Transcription/Translation System (Promega). The T7 promoter sequence was added 5’ to all forward primers to allow transcription.

**Table 1:** 

Fragments (Fr)	Primers
Fr1	Fw 5’-TAATACGACTCACTATAGGGATGTCCACAAACTCTGGAGCATCT-3’
	Rv 5’-TGCCTATTGCTCGACTTTTG-3’
Fr2	Fw 5’-TAATACGACTCACTATAGGGATGGGTCCTGCCTTTATTCA-3’
	Rv 5’-TTTCGTATTGGGCTGAGTGA-3’
Fr3	Fw 5’-TAATACGACTCACTATAGGGATGACCTCTCCTCGAGTGGTAGTCA-3’
	Rv 5’-ACACCACCCCTGGTGAAACT-3’
Fr4	Fw 5’-TAATACGACTCACTATAGGGATGAATAAGCCCCCTGCAGTTTC-3’
	Rv 5’-TGCTGAATATTCTCGGTTTCTACA-3’
Fr5	Fw 5’-TAATACGACTCACTATAGGGATGGTAGAAACCGAGAATATTCAGCAC-3’
	Rv 5’-GCAGCATCATCAGATCCCATA-3’
Fr6	Fw 5’-TAATACGACTCACTATAGGGATGCCACTGCTTACTGGCTTATCG-3’
	Rv 5’-ATAAAGGCAGGACCCATGCT-3’

### Cell lines and treatments

Tamoxifen-resistant cells were obtained by continuous passage of MCF7 in the presence of sub-lethal doses of Tamoxifen [16]. For the experiments, we used two independent subcultures from MCF7/TAMR-4 (independently passaged >15 times), here indicated as TAMR-4.1 and TAMR-4.2, and the MCF7/TAMR-8 cell line, indicated as TAMR-8. Resistant cells, collectively called TamR, were continuously propagated in phenol red-free DMEM/F12 1:1 (Life Technologies), supplemented with 1% FBS (Biochrom, S0115-1) and 10^−6^ M 4OHT. For proliferation assays, TamR cells were plated at 1 x 10^4^ cells/well in 96-well plates in phenol red-free DMEM + 10^−6^ M 4OHT in the absence of FBS, then were treated for 24 hours with the appropriate concentrations of peptides, as described below. Proliferation was measured by two-hours bromo-deoxyuridine incorporation (Cell Proliferation Biotrak ELISA System kit, GE-Healthcare, RPN20) followed by chemi-luminescence detection on a Bio-Rad Benchmark plus spectrophotometer.

### Cell transfection

HEK293T cells were plated at 15 x 10^5^ cells/plate in 100*20mm plates and after 18 hours later transfected with either i) 6 μg pCMV-T7-Tab2(406–531) and 6 μg pCMV-p65-ERα(1–60); or ii) 6 μg pCMV-T7-Tab2(406–531) and 6 μg p3XFLAG-CMV-ERα full-length; or iii) 6 μg pCMV-T7-Tab2(406–531) and 6 μg p3XFLAG-CMV-AR full-length, using LipofectAMINE2000 (LifeTechnologies) according to the manufacturer's instructions. After 4 hours, the medium was changed to high glucose DMEM supplemented with 10% FBS and cells harvested 24 hours later.

### Treatment with peptides

The TAT-Tab2-pept3 or scrambled TAT-Tab2-pept3 peptides, at concentrations ranging from 1 μM to 100 μM, were added to TamR cells in phenol red-free DMEM + 10^−6^ M 4OHT in the absence of FBS, due to the low stability of the peptide in serum. After 1 hour, 1% DC-FBS was added.

### Co-immunoprecipitation, pull-down and immunoblotting

Co-immunoprecipitations of ERα and Tab2 or AR and Tab2 were carried out from total cell lysates of HEK293T cells. HEK293T cells were lysed with RIPA Buffer (150 mM NaCl, 1% NP-40, 0.5% sodium deoxycholate, 0.1% SDS, 50 mM Tris-HCl pH 7.4, 2 mM EDTA, 50 mM NaF, 1X PIC, 1 mM PMSF) put on ice 30 min and centrifuged at 12,000xg for 10 min. Supernatants were diluted in RIPA Buffer without SDS. 15 μg of anti-NFkB-p65 antibody or 15 μg of anti-epitope T7 antibody were used. The samples were incubated overnight at 4°C, then 50 μL of protein A sepharose beads in RIPA Buffer without SDS were added, and incubation continued for another 2 hours. The pellet was washed 1 time in LiCl Buffer (0.25 M LiCl, 1% NP40, 1% Na DOC, 1mM EDTA, 10mM Tris-HCl pH8), 1 time in RIPA Buffer without SDS and 2 times in CoIP Buffer (20 mM Hepes pH 7.9, 10% glycerol, 5 mM MgCl2, 0.2 mM EDTA, 1 mM DTT, 0.1% NP-40, 1 mM PMSF, 50 mM NaF, 1X PIC). The samples were analyzed by immunoblotting with the appropriate antibodies.

**Fig 1:**
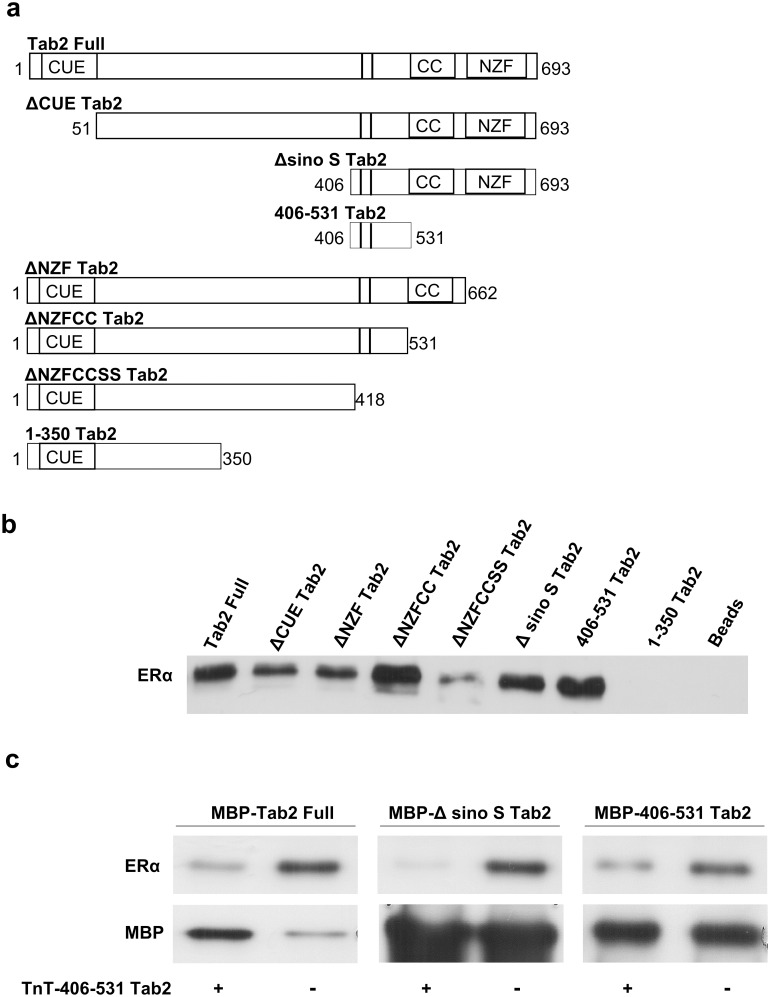
The central domain of Tab2 contains the major determinants of Tab2/ERα interaction. a. Scheme of Tab2 fragments of different length expressed in bacteria as MBP fusion proteins. The functional domains are indicated as follows: the CUE domain, the CC (coiled coil) domain including the nuclear export sequence, the NZF (novel zinc finger) domain and the sequence (II) containing two phosphorylation sites (S419 and S423). **b**. MBP pull-down assays, using ERα-overexpressing HEK293T cell lysate and MBP-Tab2 fusion proteins described in a). The loading control for all Tab2 fragments is provided in S1 Fig. **c**. MBP pull-down assays using the MBP fusion proteins Tab2 full-length, ΔsinoSTab2 and Tab2_(406–531)_ as a control, and ERα-overexpressing HEK293T cell lysates in the presence or not of the *in vitro* transcribed and translated Tab2_(406–531)_ fragment. The lower bands represent the total amount of MBP-Tab2 fusions present in the assay.

For in vitro pull-down assays, purification of MBP-fusion proteins (MBP-Tab2 full-length, MBP-Δ CUETab2, MBP-ΔsinoSTab2, MBP-406-531Tab2, MBP-ΔNZFTab2, MBP-ΔNZFCCTab2, MBP-ΔNZFCCSSTab2, and MBP-1-350Tab2) and GST-ERα (1–45) protein were performed according to manufacturers’ protocol (General Healthcare). Pull-down assay described in Fig 1b was done using MBP-Tab2 proteins and ERα-overexpressing HEK293T cell lysates. HEK293T cell lysate was prepared by adding 300 μl of Lysis Buffer 1X (150 mM NaCl, 2 mM Tris/HCl pH 7.8, 2 mM EDTA, 0.5% NP-40, 2 mM Na3VO4, 1X PIC, 1 mM PMSF). MBP-Tab2 fusion constructs (10 μg) were incubated in pull-down buffer (125 mM NaCl, 20 mM Tris/HCl pH 7.8, 10% glycerol, 0.1% NP-40, 0.5 mM DTT, 1X PIC, 1 mM PMSF) with HEK293T cell lysate for 1.5 hours at 4°C under rotation. A volume of 50 μl of prewashed Amylose Resin beads was then added for 1 hour at 4°C on rotation.

Pull-down assay described in Fig 1c was performed using MBP-Tab2 proteins, in vitro translated and transcribed Tab2(406–531) and ERα-overexpressing HEK293T cell lystes. 10 μg MBP-Tab2 fusion constructs (MBP-Tab2 full-length, MBP-ΔsinoSTab2 and MBP-Tab2(406–531)) were preincubated in pull-down buffer with 5 μl in vitro translated and transcribed Tab2(406–531) for 1 hour on rotation at 4°C and then mixed with HEK293T cell lysate for 1.5 hours at 4°C under rotation. A volume of 50 μl of prewashed Amylose Resin beads was then added for 1 hour at 4°C on rotation.

**Fig 2:**
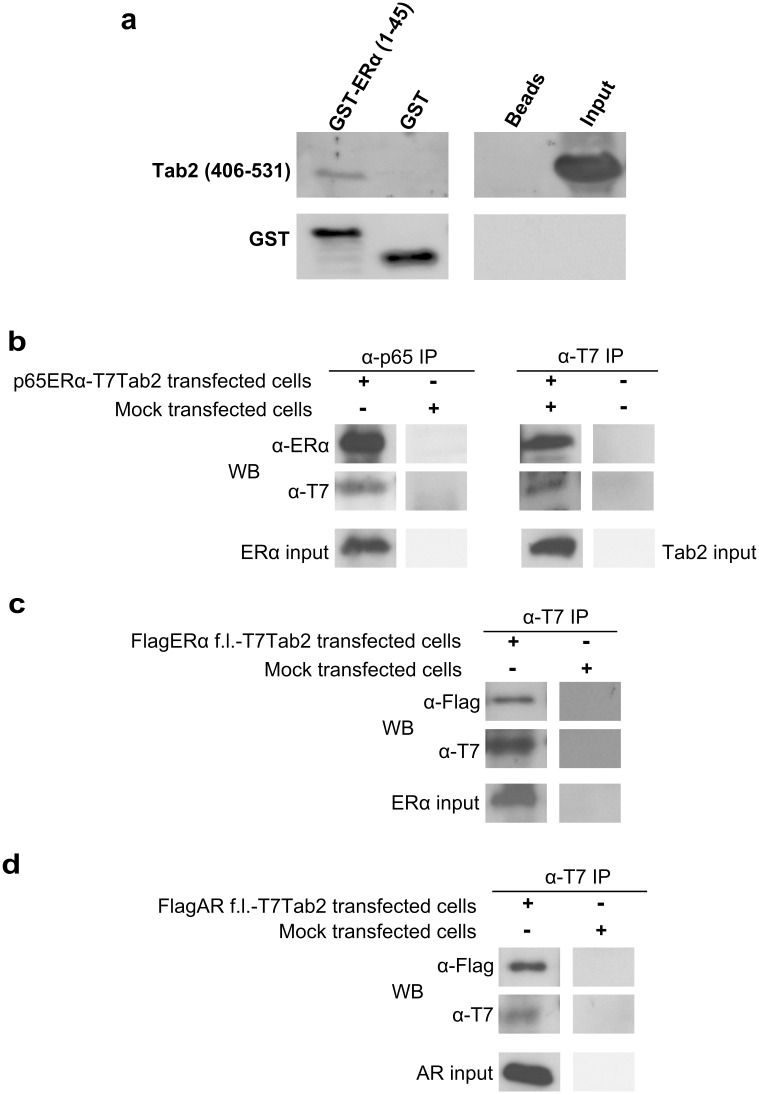
The central domain of Tab2 interacts with the conserved N-terminal domain of ERα and AR. **a**. GST pull-down assay using the ERα fragment encompassing aa 1–45 expressed as GST fusion protein and the *in vitro* transcribed and translated Tab2_(406–531)_. **b,c,d**. Co-immunoprecipitation of T7-tagged Tab2_(406–531)_ and (**b**) p65-tagged ERα_(1–60)_; (**c**) Flag-tagged full-length ERα; (**d**) Flag-tagged full-length AR. The plasmids were transiently overexpressed in HEK293T cells, as follows: p65-ERα_(1–60)_ plus T7-Tab2_(406–531)_; Flag-full-length ERα (f.l.) plus T7-Tab2_(406–531)_; Flag-full-length AR (f.l.) plus T7-Tab2_(406–531)_. Mock transfected cells are untransfected cells. Anti-p65 and anti-T7 immunoprecipitates were carried out from total cell lysates and were analyzed by western blot with anti-ERα, anti-Flag and anti-T7 antibodies.

Pull-down described in Fig 2a was done using GST-ERα (1–45) and in vitro translated and transcribed Tab2(406–531). 10 μg GST-ERα (1–45) or GST alone were incubated in pull-down buffer with 5 μl *in vitro* translated and transcribed Tab2(406–531) for 2 hour on rotation at 4°C. A volume of 50 μl of prewashed Glutathione Resin beads was then added for 1 hour at 4°C on rotation.

**Fig 3:**
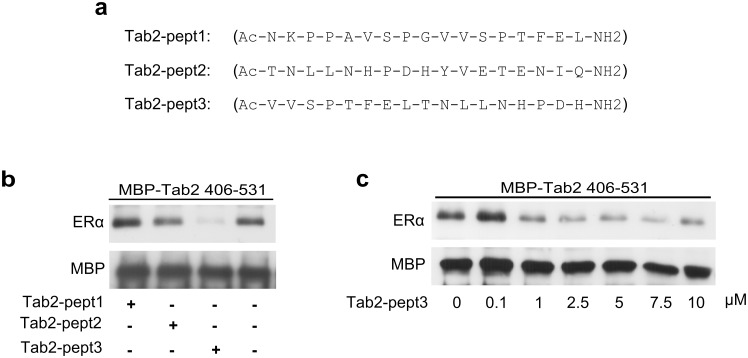
A Tab2 mimic peptide displaces the Tab2/ERα interaction. **a**. Aminoacid sequences of the three synthetic partially overlapping 17-aa each peptides, corresponding to different portions of the Fragment 4 described in S2 Fig. (here called Tab2-pept1, Tab2-pept2 and Tab2-pept3). **b**. MBP pull-down assays, using MBP-Tab2_(406–531)_, recombinant hERα (1 nM) and the three different synthetic Tab2 peptides (at a concentration of 10 μM each). **c**. Dose-response curve on pull-down assays, using MBP-Tab2_(406–531)_, recombinant hERα (1 nM) and the Tab2-pept3 at concentrations ranging from 0 to 10 μM.

Pull-down assays described in Fig 3a and 3b were performed using MBP-Tab2(406–531), Tab2 peptides and recombinant hERα. 10 μg MBP-Tab2(406–531) were preincubated in pull-down buffer with 10 μM each Tab2-pept1, Tab2-pept2, Tab2-pept3 or with increasing concentration (100 nM to 10 μM) of Tab2-pept3 for 1 hour on rotation at 4°C and then mixed with recombinant hERα (1 nM) for 1.5 hours at 4°C under rotation. A volume of 50 μl of prewashed Amylose Resin beads was then added for 1 hour at 4°C on rotation.

In all pull-down assays, the protein complexes were washed 3 times with pull-down buffer, recovered by centrifugation at 12,000xg, resuspended in SDS-gel loading buffer and analyzed by immunoblotting with the appropriate antibodies.

### Statistical analysis

Single-point data significance was evaluated using T-test for paired, two-tailed statistics. For dose-response curves, a generalized least square regression model was used to ascertain whether a significant interaction occurred between wild type/scrambled peptides and time, with respect to cell proliferation. A correlation structure was specified to account for measures at different dose levels over repeated experiments. A continuous-time autoregressive of order 1 (CAR1) correlation structure resulted the best model fit, based on AIC values. Non-linear dose-response relationship was tested using Wald test.

## Results

### The central domain of Tab2 contains the major determinants of Tab2/ERα interaction

Interaction of ERα with a Tab2 fragment comprising aa. 406–531 (Tab2_(406–531)_) was shown previously [8]. However, we wanted to evaluate if this “central” domain contains the major determinants of this interaction. In order to obtain this information, we designed a series of fragments of Tab2 of different length containing, or deleted of, the following functional domains: the CUE domain (aa 8–50); the CC domain (aa 530–615) including the nuclear export sequence; the NZF domain (aa 663–693); and the sequence containing the two Serines (S419 and S423) identified as phosphorylation sites for MEKK1 [5] (Fig 1a). These fragments were expressed in bacteria as MBP fusion proteins and tested in pull-down assays. We observed that full-length Tab2, as expected, and all the fragments retaining at least part of the C-terminus, up to the critical regulatory MEKK1 phosphorylation sites (S419 and S423), pulled-down in a very efficient manner ERα from lysates of overexpressing HEK293T cells, whereas mutants lacking this domain did not (Fig 1b). Loading controls for this experiment are provided as S1 Fig. To further confirm the importance of this domain, we constructed a fragment containing only the central part of Tab2 (Tab2_(406–531)_), which showed strong interaction with ERα (Fig 1b). Next, we expressed Tab2_(406–531)_ by *in vitro* transcription and translation and used it in competition assays including ERα from lysates of overexpressing HEK293T cells and MBP fusion proteins Tab2 full-length, ΔsinoSTab2 and itself as a control (Fig 1c). Even though the MBP fusions were quite different in the different conditions, we can conclude that Tab2_(406–531)_ is very efficient in competing these interactions. Taken together, these results confirm that the domain of Tab2 spanning aa. 406–531 is likely to contain the major determinants of ERα interaction.

### The central domain of Tab2 interacts with the conserved N-terminal domain of ERα and AR

Next, we asked whether the fragment of ERα encompassing aa 1–45, containing the conserved HX7LL motif that causes recruitment of Tab2 [7,8] was sufficient to mediate interaction with Tab2_(406–531)_. ERα_(1–45)_ was expressed in bacteria as GST fusion protein and Tab2_(406–531)_ was *in vitro* transcribed and translated. As shown in Fig 2a, we observed that the ERα N-terminal fragment (aa 1–45), but not GST alone, was sufficient to pull-down the Tab2 central domain.

One important point is whether this interaction exists *in vivo*. To ascertain this, we overexpressed T7-tagged Tab2_(406–531)_ and p65-tagged ERα_(1–60)_ in HEK293T cells and carried out anti-T7 and anti-p65 immunoprecipitation from total cell lysates. We observed that T7-Tab2_(406–531)_ efficiently co-immunoprecipitated ERα_(1–60)_, and vice-versa (Fig 2b), confirming the interaction between the central domain of Tab2 and the N-terminal region of ERα also in living cells. The same experiment was carried out by overexpressing in HEK293T cells the Flag-tagged full-length ERα: as shown in Fig 2c, also in this case the interaction was clearly demonstrated.

The HX7LL motif present in the N-terminus of ERα is conserved in other steroid receptors, among which PgR and AR, but not ERβ [7]. Therefore, we asked whether the central domain of Tab2 was also able to interact with other receptors, specifically with AR that plays a role in the response/resistance of prostate cancer cells to anti-androgenic drugs, in analogy to anti-estrogens in breast cancer cells. To achieve this goal, we overexpressed T7-tagged Tab2_(406–531)_ and Flag-tagged full-length AR in HEK293T cells and carried out anti-T7 immunoprecipitation from total cell lysates. We observed that T7-Tab2_(406–531)_ co-immunoprecipitated full-length AR (Fig 2d). Thus, the central domain of Tab2 is responsible of the interaction of Tab2 with steroid receptors in general.

### A Tab2 mimic peptide displaces the Tab2/ERα interaction

Taking advantage of the competition of *in vitro* transcribed and translated Tab2_(406–531)_ in Tab2/ERα interaction (Fig 1c), we further narrowed down the essential interaction motif using a pull-down competition screening. To achieve this goal, 6 partially overlapping fragments were designed on the sequence of the Tab2 central domain (S2b Fig), produced by PCR and *in vitro* transcribed and translated. Competition of these peptides with Tab2/ERα interaction was evaluated in pull-down assays using the recombinant protein MBP-Tab2_(406–531)_ and recombinant hERα (1 nM). The best competing fragment in this assay was Fragment 4 (S2a Fig) representing aminoacids 471–504. This peptide also contains an LTNLL motif, structurally interesting because potentially able to create an amphipathic α-helix that can be responsible of the interaction (S2 Fig). On the basis of these results, three partially overlapping 17-aa synthetic peptides, corresponding to different portions of the above described Fragment 4, thereafter called Tab2-pept1, Tab2-pept2 and Tab2-pept3, were synthesized (their sequences are reported in Fig 3a). MBP pull-down assays, using MBP-Tab2_(406–531)_ and recombinant hERα (1 nM) were used to assay for the ability of these peptides to compete out the interaction. As shown in Fig 3b, we observed that the synthetic Tab2-pept3, but not Tab2-pept1 nor Tab2-pept2, was able to compete out efficiently the in vitro Tab2/ERα interaction. This data indicate that the interacting motif is comprised within, or partly overlapping to, these 17-aa. Interestingly, this peptide includes the LTNLL motif discussed above. A dose-response curve demonstrated that Tab2-pept3 is already efficient in displacing the interaction at a concentration of 1 μM (Fig 3c).

### A 17-aa Tab2 mimic peptide reduces the growth of MCF7 TamR cells in the presence of Tamoxifen

**Fig 4:**
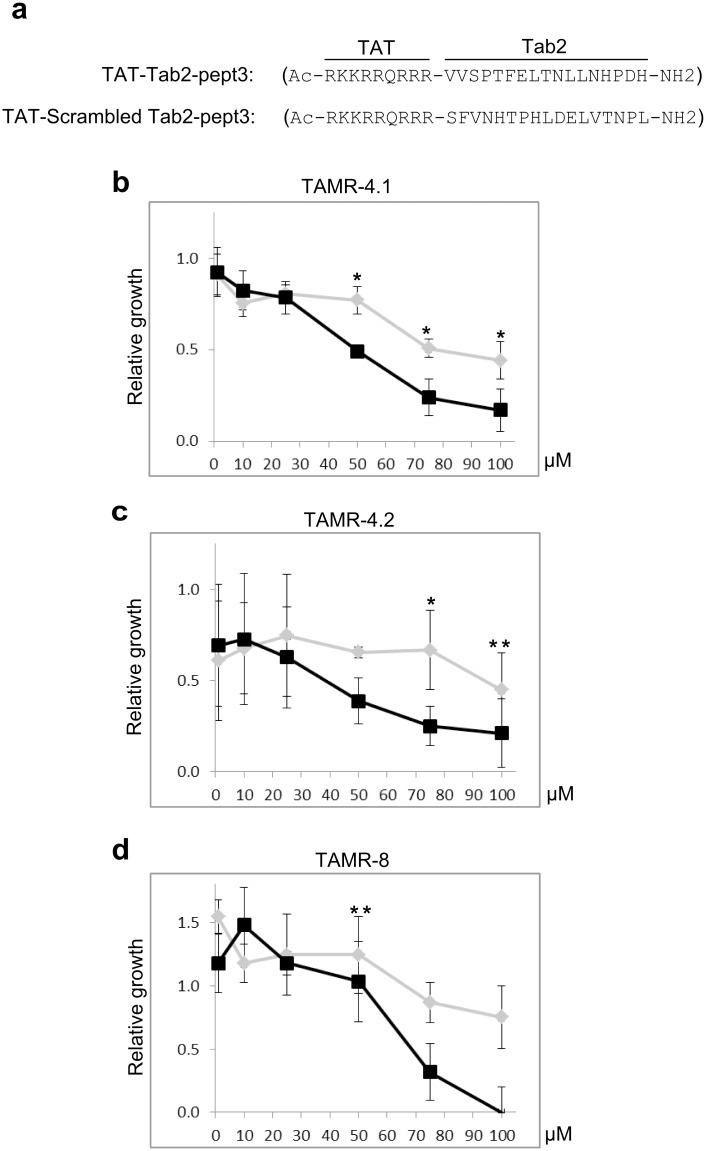
A 17-aa Tab2 mimic peptide reduces the growth of MCF7-TamR cells in the presence of Tamoxifen. **a**. Aminoacid sequences of Tab2-pept3 fused N-terminally to the minimized carrier sequence of the viral TAT protein, and the scrambled version. **b**. MCF7-TamR cells were treated with increasing concentration (1 to 100 μM) of TAT-Tab2-pept3 (∎) or its scrambled version (⧫) in absence of serum. After 1 hour, 1% DC-FBS and 10^−6^ M 4OHT were added and the effect on cell proliferation was measured 24 h later. Data are means ± s.d. of triplicate experiments. Stars refer to significance of individual concentration points, using T-test (* p<0.05; ** p<0.01). Continuous-time autoregressive model statistics gave p<0.02 for TAMR-4.1 (b); p<0.09 for TAMR-4.2 (c); p<0.08 for TAMR-8 (d).

Finally, we addressed the question whether such interfering peptide could relieve Tab2 inhibition of Tam response in cultured TamR cells, as we described for the ERα-derived peptide [8]. We fused Tab2-pept3 N-terminally to the minimized carrier sequence of the viral TAT protein, thus obtaining a cell permeable 26-aa peptide, called TAT-Tab2-pept3, and the corresponding scrambled version (their sequences are shown in Fig 4a). To this purpose, we used three different subcultures of clones of MCF7 TamR cells [16], in which Tam resistance was shown to be dependent, at least in part, on Tab2 constitutive phosphorylation [8]. Cells were treated with increasing concentrations (1 to 100 μM) of TAT-Tab2-pept3, or its scrambled version, for 1 h in the absence of serum, then serum was added back and the effect on cell proliferation was measured 24 h later. Results of these experiments are shown in Fig 4b–4d. The effect of peptide treatment was slightly different between TamR clones (TAMR-4 and TAMR-8) and also among subcultures of the same clone (TAMR-4.1 and -4.2). TAT-Tab2-pept3 decreased the growth of cells in the presence of 1 μM 4OHT in a dose-dependent fashion, with estimated LD50 between 50–70 μM. The scrambled peptide also showed some inhibitory effect at the highest concentrations, possibly due to toxic effect. Using a continuous-time autoregressive model we observed a significant response in TAMR-4.1 (p<0.02), while in other cell lines there was marginal significance. T-test analysis demonstrated at least one concentration point with significant effect (see Fig 4 legend). The TAT-Tab2-pept3 had no effect on the growth of cells cultured in absence of 4OHT (data not shown). These results further demonstrate that interfering Tab2/ERα interaction may be exploited to revert in part Tamoxifen resistance in breast cancer cells *in vitro*.

## Discussion

The data presented here suggest that a central domain of Tab2 contains the major determinants of the interaction with steroids receptors, thus providing a further annotation to this multifunctional protein. We present evidence that a peptide mimicking aa. 480–496 of Tab2 is able to compete efficiently with Tab2/ERα interaction *in vitro*, thus narrowing down the interacting domain to a smaller region containing this motif (SRID = steroid receptor interacting domain). In addition, we show that the corresponding cell-permeable peptide, TAT-Tab2-pept3, reduces the growth of Tam-resistant breast cancer cells, cultured in the presence of Tamoxifen, in a dose-dependent fashion. Thus, TAT-Tab2-pept3 together with the ERα-TAT peptide previously identified [8] represent lead compounds to reverse Tam-resistance in breast cancer cells. Moreover, considering that the central domain of Tab2 also interacts with AR (and most likely with other steroid receptors sharing the same N-terminus), it represents a promising tool for further pharmacological developments.

The SRID was not previously identified as a functional domain. It is embedded in a region flanked on the C-terminal side by the CC-domain, which is supposed to provide a quite rigid structure, and presenting a number of experimentally proven phosphorylation sites. In addition, within the minimal competing peptide (Tab2-pept3) the LTNLL motif may justify formation of an amphipathic α-helix that is frequently found in interacting protein surfaces. In order to discuss the potential significance of the SRID, we have to consider the proposed molecular mechanisms underlying Tab2-mediated derepression [7,17]. In this context, Tab2 is recruited to genes that are repressed following antagonist treatment (either 4OHT in the case of ERα or Bicalutamide in the case of AR) by direct interaction with the N-terminal domain of steroid receptors. Since ERα/AR are antagonist-bound, they interact with NCoR rather than coactivators [18,19]. Apparently, Tab2 is not necessary to this interaction, since its experimental down-regulation does not affect gene response to these drugs [7]. In the presence of signals activating Tab2, i.e. either inflammatory signals or Receptor Tyrosine kinase activation or other unidentified reasons as in TamR cells [8], Tab2 unveils its NES, dismisses interaction with the steroid receptors, and translocates to the cytoplasm in complex with NCoR. Thus, the Tab2/ERα interaction should be resolved following phosphorylation and parallels NES unmasking. In this context, it is interesting to note that both the phosphorylation sites and the NES are located very close to the identified SRID.

Tab2 NES (LQRELEI) is located in the CC region between aa 547–561. Site-directed mutagenesis of the NES abrogated Tab2 translocation to the cytoplasm following phosphorylation in response to IL-1β, also blocking NCoR export [5]. In the region spanning aa. 350 to 590, several Ser/Thr phosphorylation sites are present [5,9–11,20] (as reported by the PhosphoSitePlus resource www.phosphosite.org) and a particularly dense cluster is present between aa 413 and 484, that are phosphorylated following activation of kinase cascades involving MEKK1 and p38, in response to cytokines and EGF. Ser419 and Ser423 are required for Tab2 export following IL-1β [5,7]. It is relevant to note that Ser482 and Thr484 are included in the SRID (Fig 1a), even though they were not identified as IL-1β-induced phosphorylation sites in the previously cited study.

Given the density of phosphorylation sites around the SRID and the proximity of the NES, it is tempting to speculate that phosphorylation may induce a conformational change in this region, leading to steroid receptor dissociation and NES unmasking. In this context, it is important to note that we did not get a “all-or-none” pattern in our mutants, suggesting that other regions of Tab2 may be involved in steroid receptor binding, perhaps by stabilizing the structure of this central domain.

In the model of antagonist-bound AR or ERα, Tab2 may bind the steroid receptor and NCoR at the same time. The interaction between Tab2 and NCoR has not been finely mapped: it involves the repressor domain I of NCoR and the region of Tab2 comprising aa 1–628 [5,6], i.e. all the protein with exclusion of the C-terminal NZF/zf-RanBP domain. Using the same *in vitro* biochemical assay performed to study the Tab2/ERα interaction, and exploring all Tab2 fragments used in this work, we observed that the domain of Tab2 involved in NCoR binding is proximal to its C-terminal region but outside the central domain interacting with ERα (data not shown), thus allowing the hypothesis of contemporaneous complex.

Although this was not the primary objective of our work, we attempted a preliminary characterization of the activity of Tab2 SRID in the context of antagonist drug resistance, in analogy to previously published data [8]. We obtained evidence that a cell-permeable peptide, TAT-Tab2-pept3, that effectively competes with Tab2/ERα interaction *in vitro*, restores Tam response in TamR breast cancer cells in a dose-dependent manner. Similar results were obtained by treating the TamR cells with a peptide version containing carrier sequence of the viral TAT protein at the C-term of the peptide (Tab2-pept3-TAT) (data not shown). It is important to note that the growth reduction of TamR cells in the presence of Tam, is observed at high concentrations of the peptide (75–100 μM) and further experiments will be necessary to define the limits between effectiveness and toxicity of this peptide.

In conclusion, we have further refined the Tab2/ERα interaction, providing structural data that can be exploited as a potential drug target for overcoming Tam resistance in breast cancer cells or translated to other contexts in which activation of the inflammatory pathway cross-talks and interferes with the response to steroid hormones.

## Supporting Information

S1 Fig Loading control for all Tab2 fragments used in MBP pull-down assays showed in Fig 1b. All Tab2 fragments are MBP fusion proteins, thus the western blot was analyzed with anti-MBP antibody. The stars (*) denote bands corresponding to the Tab2 fragments indicated at the top of the lanes.
(TIF)

S2 Fig Pull-down competition screening of Tab2(406–531) fragments. **a.** The recombinant protein MBP-Tab2_(406–531)_ and recombinant hERα (1 nM) in the presence or not of the *in vitro* transcribed and translated 6 partially overlapping fragments on the sequence of the Tab2 central fragment 406–531 were used. Input = loading control for recombinant hERα. **b.** Aminoacid sequences of the 6 partially overlapping fragments designed on the sequence of Tab2_(406–531)_. In fragment 4 the LTNLL sequence is shown in bold. Displacing activity = ability in interfering in Tab2/ERα interaction.
(TIF)

S3 Fig Original blots for Fig 1b. (TIF)

S4 Fig Original blots for Fig 1c. (TIF)

S5 Fig Original blots for Fig 2a. (TIF)

S6 Fig Original blots for Fig 2b, 2c and 2d. (TIF)

S7 Fig Original blots for S2a Fig. (TIF)

S8 Fig Original blots for Fig 3b. (TIF)

S9 Fig Original blots for Fig 3c. (TIF)

S10 Fig Effect of TAT-Tab2-pept3 in the presence or absence of 4OHT. MCF7-TAMR-4.2 cells were treated with 75 μM TAT-Tab2-pept3 (black bars) or its scrambled version (gray bars) in the absence of serum. After 1 hour, 1% DC-FBS plus or minus 10^−6^ M 4OHT were added and the effect on cell proliferation was measured 24 h later. Data are means ± S.D. of cell growth evaluation in pentaplicate referred to a single representative experiment.
(TIF)
