# Supplementary figures and images for: A Novel Functional Domain of Tab2 Involved in the Interaction with Estrogen Receptor Alpha in Breast Cancer Cells
Source: PLoS One. 2016 Dec 19;11(12):e0168639. doi: 10.1371/journal.pone.0168639 (PMC5167418; doi:10.1371/journal.pone.0168639)

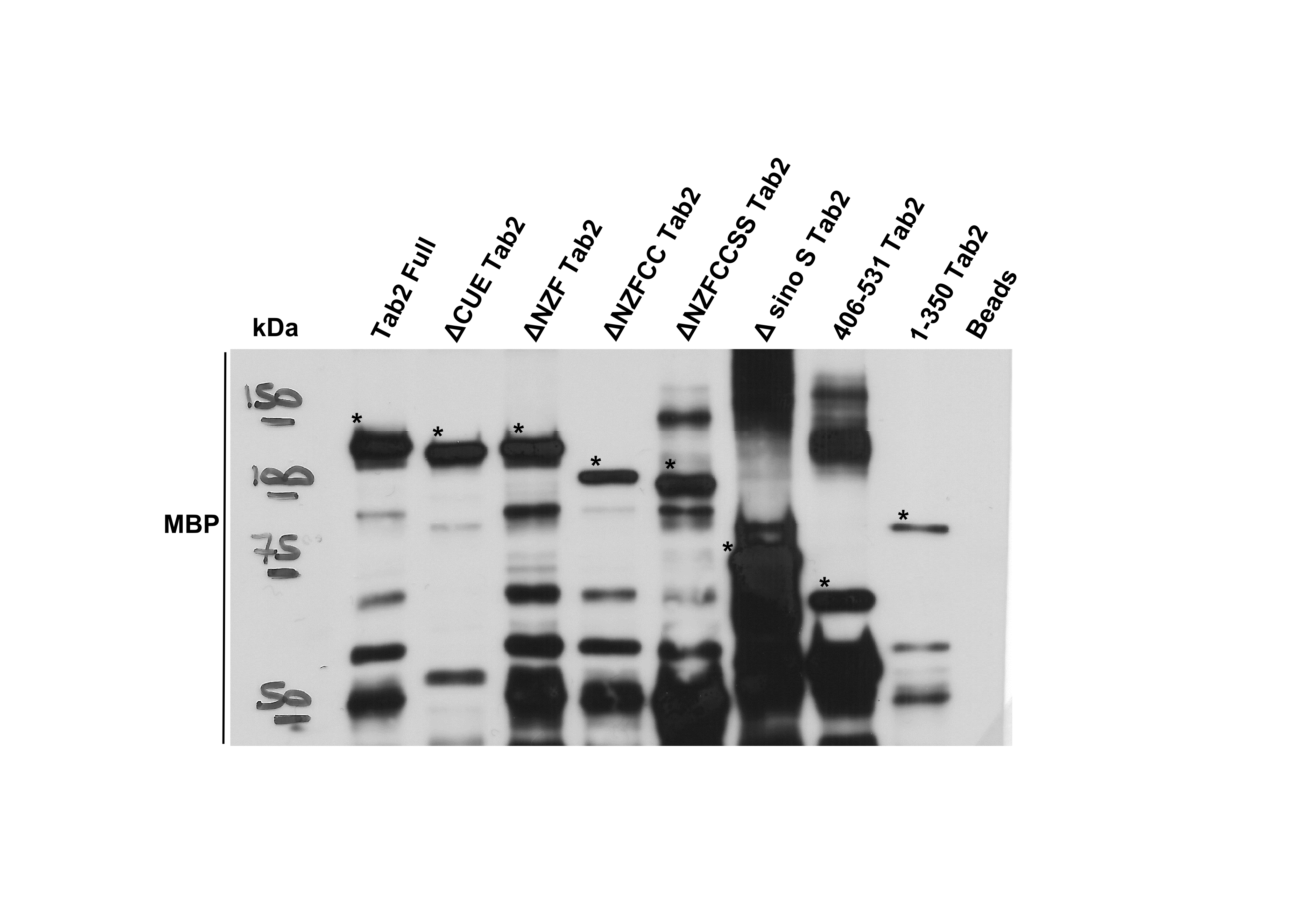

Supplement: S1 Fig — All Tab2 fragments are MBP fusion proteins, thus the western blot was analyzed with anti-MBP antibody. The stars (*) denote bands corresponding to the Tab2 fragments indicated at the top of the lanes. (TIF) [file pone.0168639.s001.tif]

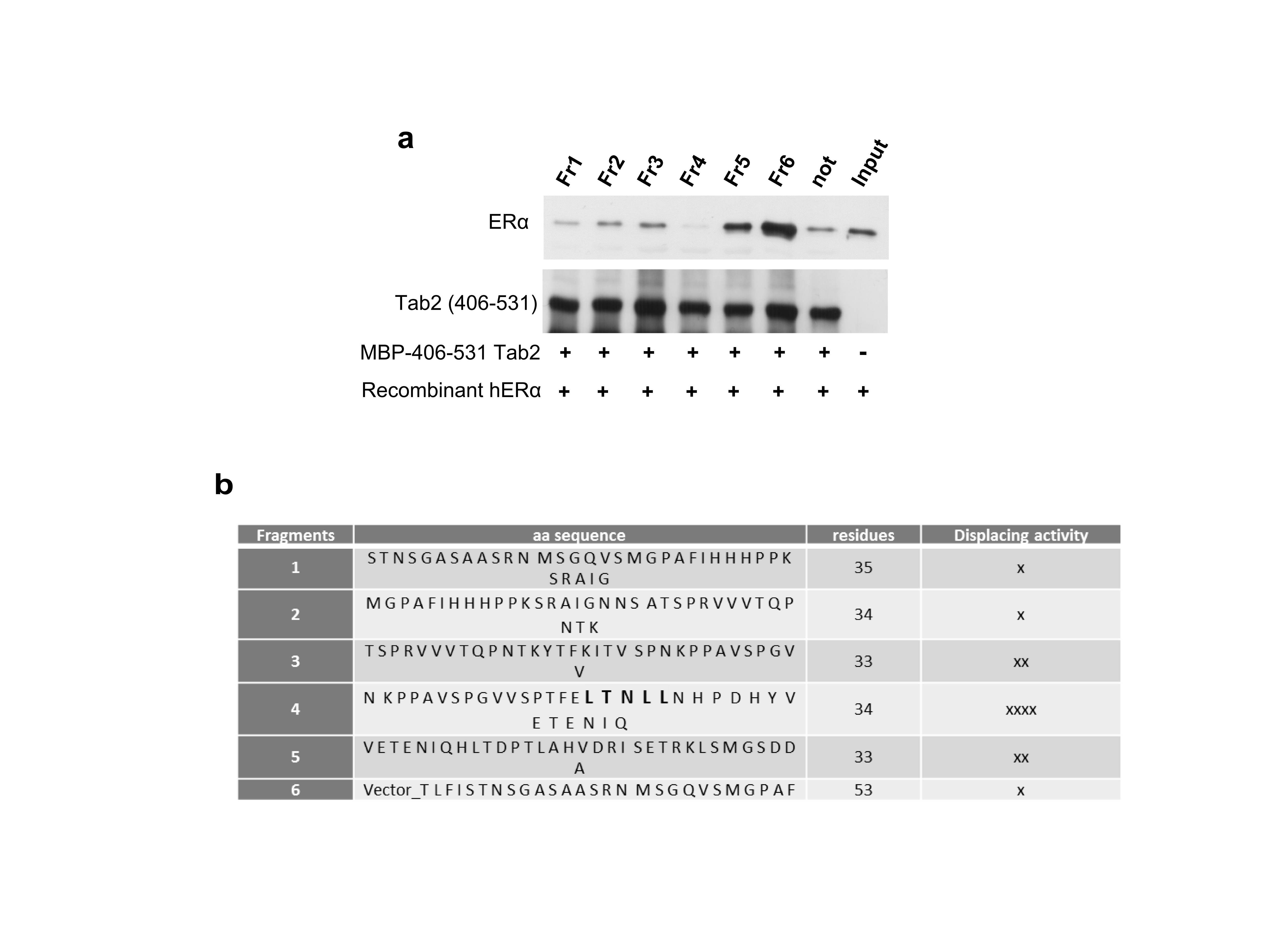

Supplement: S2 Fig — a. The recombinant protein MBP-Tab2(406–531) and recombinant hERα (1 nM) in the presence or not of the in vitro transcribed and translated 6 partially overlapping fragments on the sequence of the Tab2 central fragment 406–531 were used. Input = loading control for recombinant hERα. b. Aminoacid sequences of the 6 partially overlapping fragments designed on the sequence of Tab2(406–531). In fragment 4 the LTNLL sequence is shown in bold. Displacing activity = ability in interfering in Tab2/ERα interaction. (TIF) [file pone.0168639.s002.tif]

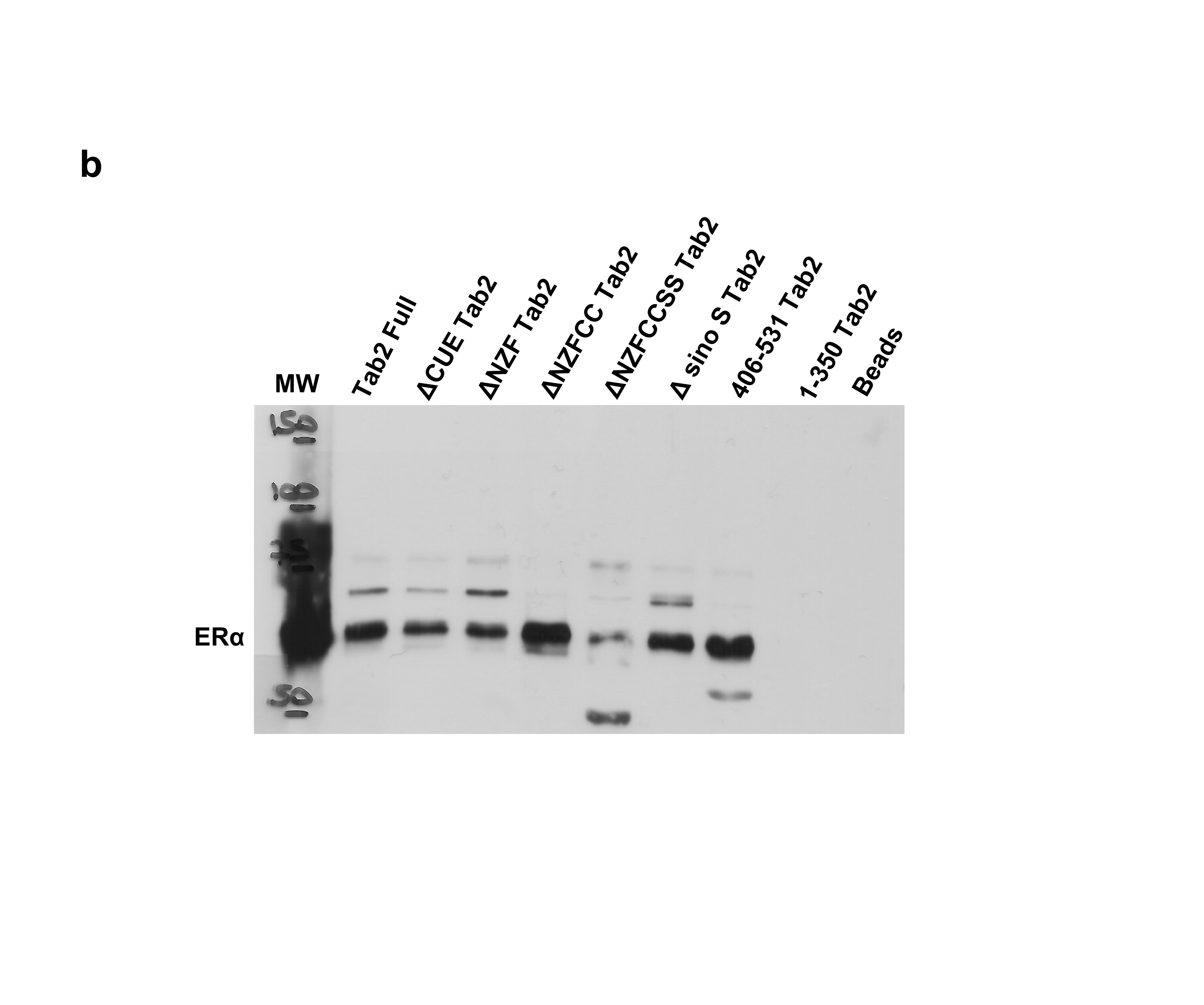

Supplement: S3 Fig — (TIF) [file pone.0168639.s003.tif]

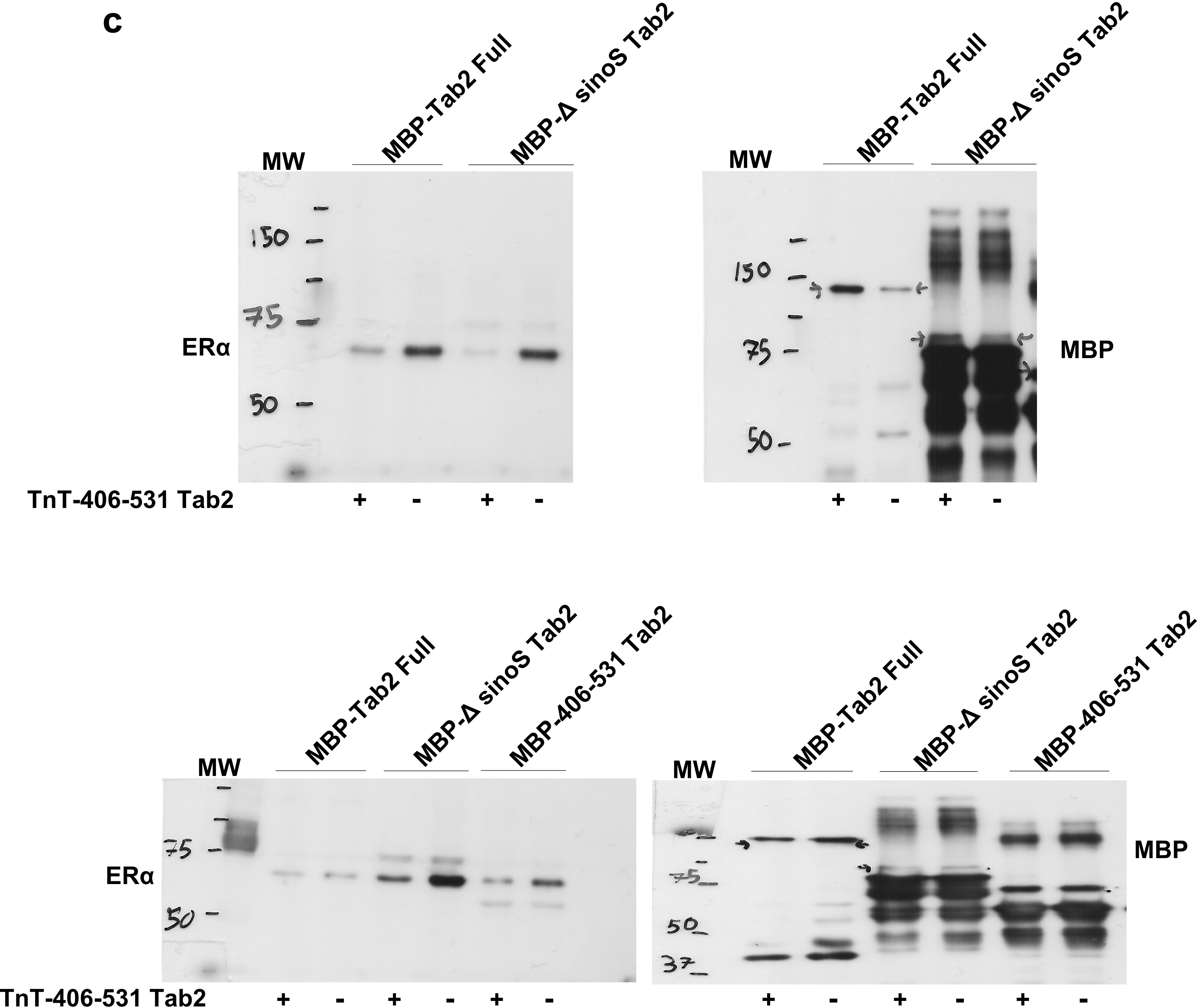

Supplement: S4 Fig — (TIF) [file pone.0168639.s004.tif]

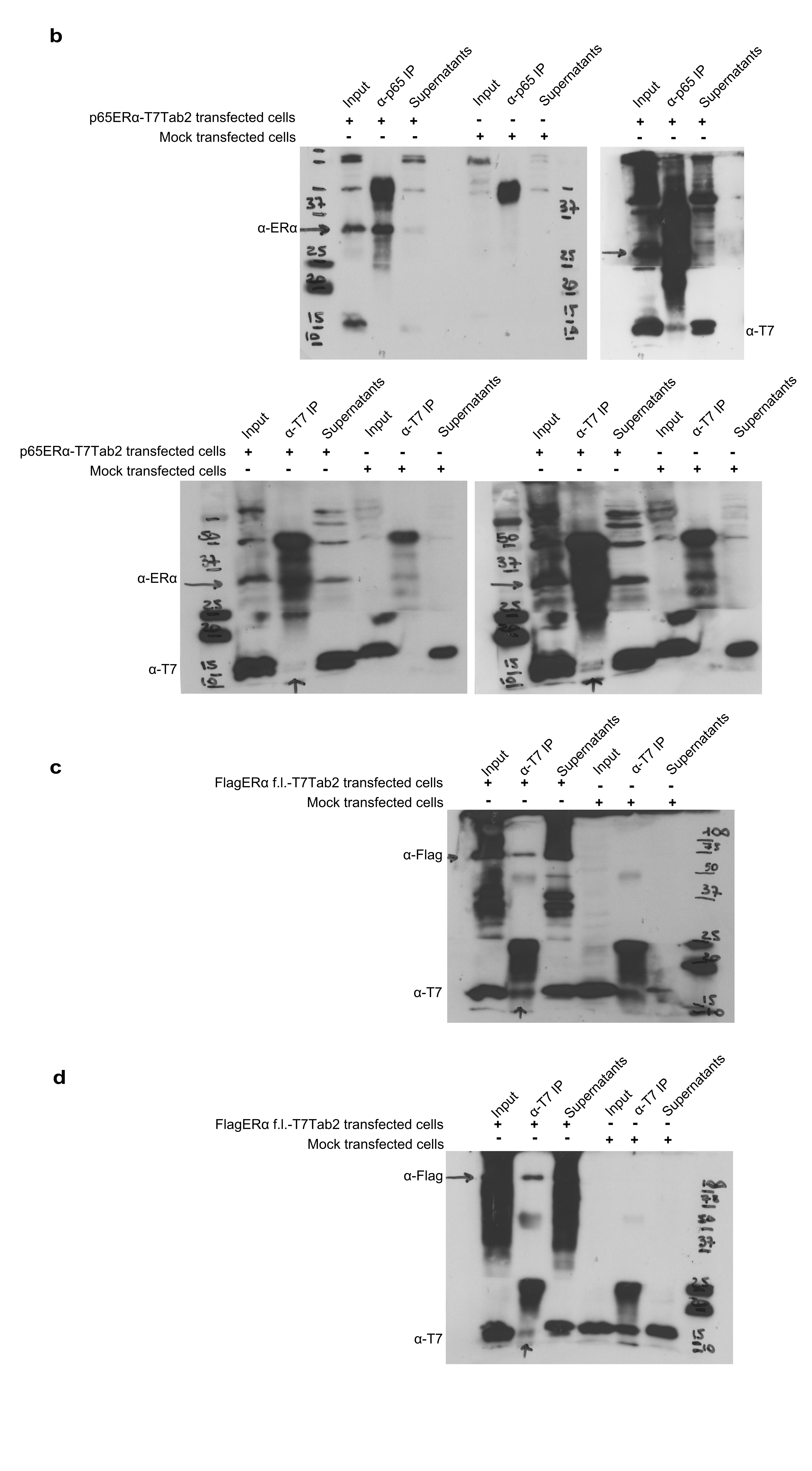

Supplement: S6 Fig — (TIF) [file pone.0168639.s006.tif]

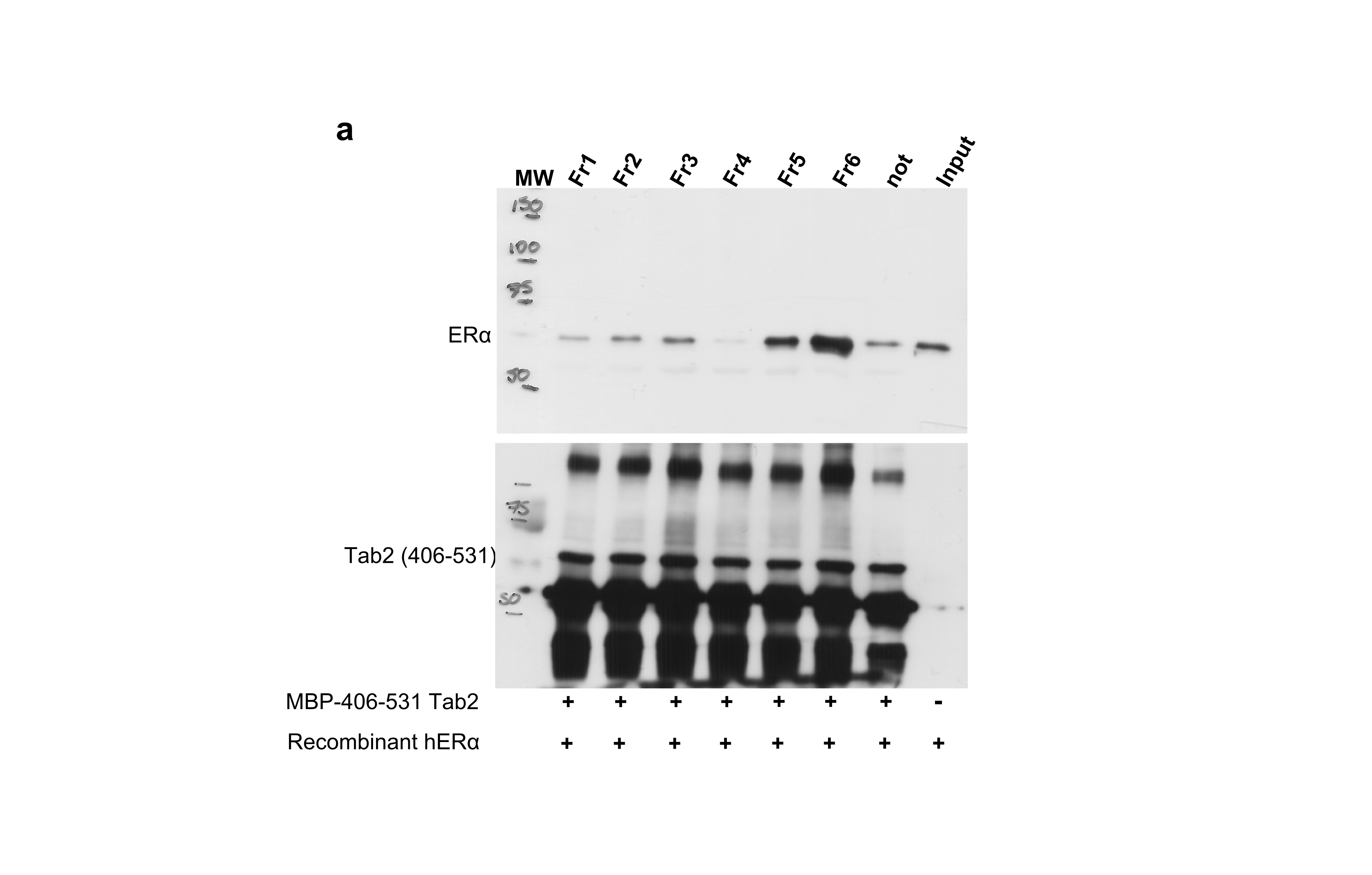

Supplement: S7 Fig — (TIF) [file pone.0168639.s007.tif]

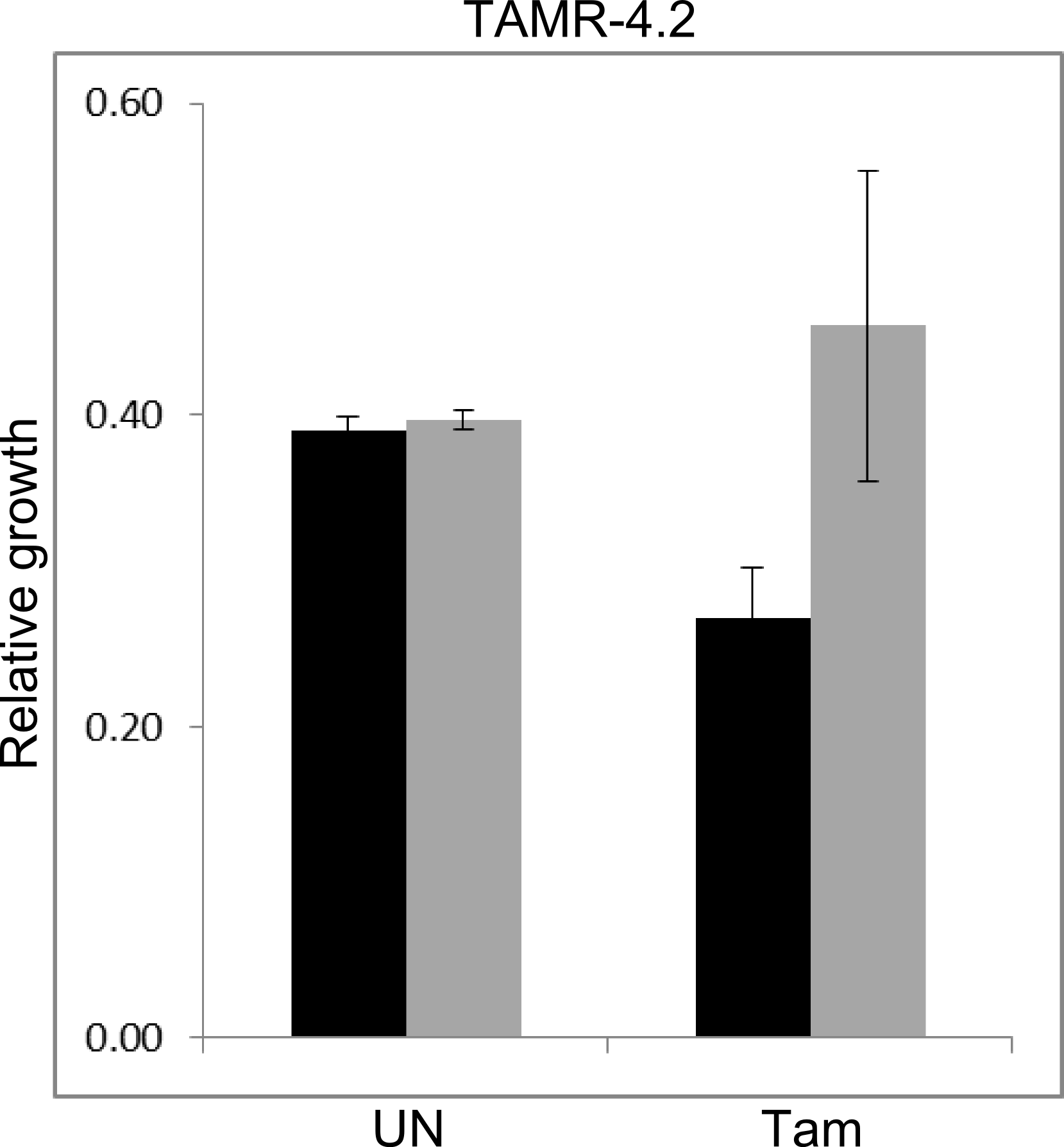

Supplement: S10 Fig — MCF7-TAMR-4.2 cells were treated with 75 μM TAT-Tab2-pept3 (black bars) or its scrambled version (gray bars) in the absence of serum. After 1 hour, 1% DC-FBS plus or minus 10−6 M 4OHT were added and the effect on cell proliferation was measured 24 h later. Data are means ± S.D. of cell growth evaluation in pentaplicate referred to a single representative experiment. (TIF) [file pone.0168639.s010.tif]
